# Complex cognition and individual variability: a mixed methods study of the relationship between creativity and executive control

**DOI:** 10.3389/fpsyg.2023.1191893

**Published:** 2023-06-22

**Authors:** Cathy J. Rogers, Andrew Tolmie, Jessica Massonnié, Michael S. C. Thomas

**Affiliations:** ^1^Centre for Educational Neuroscience, Department of Psychological Sciences, Birkbeck University of London, London, United Kingdom; ^2^Centre for Educational Neuroscience, Department of Psychology and Human Development, UCL Institute of Education, University College London, London, United Kingdom; ^3^School of Education, Languages and Linguistics, Faculty of Humanities and Social Sciences, University of Portsmouth, Portsmouth, United Kingdom

**Keywords:** creativity, executive control, mixed methods, methodology, qualitative, quantitative, triangulation

## Abstract

One of the methodological challenges of educational neuroscience is understanding real world cognition in the multifaceted environment of the classroom. Complex cognition does not simplify to *processes* (which might be satisfactorily measured in the lab) but to sets of activities, likely to vary between individuals, which involve the iterative use of multiple processes, as well as the environment, over an extended period of time. As such, studying complex cognition requires methodological flexibility; any single method is unlikely to provide complete answers. We illustrate this idea with our research exploring the relationship between executive control (EC) and creativity in primary school age children; in it, we used both qualitative and quantitative tools and a novel approach to bringing both sets of findings together. Quantitative findings helped inform ‘how much’ a participant could deploy EC or creative thinking, while qualitative findings told us more about ‘how’ they deployed EC in their creativity. Through triangulating findings, we gained insights which would have remained obscure using either approach alone; namely, first, that wide variation in how children deploy EC in creativity means that the same creative results can be achieved with very different levels of EC involvement, and second, that high levels of EC can limit creativity. We argue that, beyond the specific findings of this study, there might be useful broader methodological lessons for educational neuroscience. We also attempt to demystify mixed methods by showing that a multi-pronged approach is more feasible than many assume; for example, by using existing, familiar tools in novel ways. In our work, we redeployed well-established quantitative tests used in creativity research as stimuli for qualitative investigation. For educational neuroscience to evolve its understanding of complex cognition, we suggest it might benefit from being innovative, open-minded and ambitious in how it exploits the diversity of methodological tools available.

## 1. Introduction

This paper will present the findings of recent work examining the relationship between creativity and executive control (EC) in children. Its relevance to educational neuroscience is twofold: firstly, in the subject matter itself given the importance of both creativity and EC in children’s education and secondly, in the methodological innovations which allow exploration of individual variability, a topic that is emerging as one of the key considerations for educational neuroscience (Donati and Meaburn, 2020; Thomas et al., 2020).

In this section, we outline the importance of understanding the relationship between executive control and creativity, for example, to predict the possible impact that training EC will have on creativity. We then describe two studies, one quantitative and one qualitative, that explore the role of EC in the creative process in 4–11 year old children. We show how triangulating results yields new insights, particularly in the demonstration of wide individual variability in the role of EC in creativity. Some of the original work is necessarily abbreviated here; interested readers are referred to the fuller version in Rogers (2021).

Creativity, along with critical thinking, communication, and collaboration, has been named by the OECD one of the essential 21st century skills (Schleicher, 2011). There are many definitions of creativity and lenses through which it is viewed but all share the notion that creativity must involve producing something new (Runco and Jaeger, 2012) – also referred to as novelty, uniqueness or originality (Abraham, 2018). The standard psychological definition includes a second attribute: value (Cropley, 2000; Boden, 2004), variously defined as appropriateness (Dietrich and Kanso, 2010), relevance (Kneller, 1965), fit (Sternberg and Lubart, 1995), usefulness (Runco and Jaeger, 2012) or effectiveness (Basadur et al., 2000), since novelty alone is not enough. Creativity must demonstrate some level of originality and be appropriate to the task in hand.

In terms of how it is operationalized, there is broad agreement that both divergent and convergent thinking are involved (Guilford, 1950, 1956; Runco, 2014). Divergent processes are open, exploratory, associative and generate multiple possible ideas (Martindale, 1999; Gabora, 2010; Sowden et al., 2015) while convergent processes involve production of a single solution and are more concerned with evaluation, analysis and refinement linked to problem solving (Guilford, 1956, 1967; Sowden et al., 2015).

The mainstays of creativity research are divergent thinking (DT) tests which require participants to generate multiple answers to a given stimulus (Guilford, 1967). The most common are the Alternative Uses Test (AUT; Guilford et al., 1978) – participants generate multiple interesting, unusual alternative uses for everyday household items such as a brick, and the Torrance Tests of Creative Thinking (TTCT) (Torrance, 1966, 1972, 1974), which assess fluency, flexibility, originality, elaboration and a range of specific creative qualities (such as humor, expressiveness) in both verbal and figural domains.

Executive control (EC), also known as ‘executive functions’ (Kerr and Zelazo, 2004; Diamond, 2013), comprises a set of mental processes that allow us “to think before we act, resist temptations or impulsive reactions, stay focused, reason, problem-solve, flexibly adjust to changed demands or priorities and see things from new perspectives” (Diamond and Ling, 2016, p. 34). Executive control has profound effects on individuals’ life outcomes: better control in childhood predicts better academic achievement, and greater wealth, health, and quality of life over the entire life span (Moffitt et al., 2011).

There is some consensus that EC is comprised of three core capabilities: storing and using information in working memory (Baddeley, 1996), flexibly switching between tasks, and inhibitory control – both control of cognitive interference and self-control (Diamond, 2013). Miyake et al.’s influential 2000 study, which involved extensive testing on a host of EC tasks within this triad, found both unity and diversity: confirmatory factor analysis showed that ‘updating’ (working memory), ‘shifting’ (flexibility) and ‘inhibition’ were ‘moderately correlated but clearly separable’ (Miyake et al., 2000).

There are many tests available to assess executive control by digital or analogue means. Some of the most commonly used are the Wisconsin card sort, various versions of Stroop, anti-saccade tasks, go/no go, digit span and backwards digit span tasks, keep track tasks, Navon global/local tasks, Simon tasks, Towers of Hanoi/London, Flanker, random number generation and various dual tasks (Miyake et al., 2000). Many are used as standard tests for single EC components (e.g., Stroop for inhibitory control) but the issue of task impurity (i.e., the fact that any executive task implicates additional cognitive processes as well as the test target itself) is widely recognized (Miyake et al., 2000; Cepeda et al., 2001; Luna et al., 2004).

How do executive control and creativity relate to one another? Both are seen as desirable, but do they work in harmony or might they be antagonistic? For example, might the narrowed focus of greater control reduce awareness of more remote possibilities which might be key to creativity? The evidence is mixed.

Creative people have long been characterized as lacking cognitive and behavioral inhibition (Eysenck, 1995; Martindale, 1999). Evidence from lesion studies, neurodevelopmental conditions (e.g., ADHD) and psychopathology have found lower inhibition associated with higher levels of creativity (Carson et al., 2003; Sawyer, 2011; White and Shah, 2011; Acosta, 2014; Boot et al., 2017). In lab studies too, there is some evidence of this inverse relationship between inhibitory control and the ability to think divergently. Radel et al. (2015) used a within-subjects design to test the effects of depleting inhibition on divergent and convergent thinking tasks. They found that exposure to a high-demand inhibitory control task led to higher fluency in the AUT.

An alternative view is that inhibitory control is an essential part of divergent thinking, based on the need to block obvious, common or repeat responses which arise from spontaneous associative thinking (Beaty and Silvia, 2012; Edl et al., 2014; Benedek et al., 2014a; Mayseless et al., 2015; Cassotti et al., 2016). Some studies assessing inhibitory control using Stroop performance have reported a positive correlation between inhibition and divergent thinking performance (Groborz and Necka, 2003; Edl et al., 2014) and when Benedek and team used latent variable analysis to look at the relationship between creativity and executive control in a sample of 243 young adults (Benedek et al., 2014b), they found that both updating (working memory) and inhibition, but not shifting ability, predicted creativity scores.

Creative development is characterized by a series of ‘lumps and bumps’ (Sternberg and Lubart, 1995; Barbot et al., 2016; Runco, 2016), with discrepant levels of recovery from downturns. One of the best documented downturns (Claxton et al., 2005; Sak and Maker, 2006; Said-Metwaly et al., 2020), is the ‘fourth grade slump’ (i.e., reduced creativity in children aged 9 and 10), first identified by Torrance (1968) and still debated. Contradictory findings on this and other ‘slumps and bumps’ might be explained by differences in the tests used: a recent meta-analysis looking specifically at divergent thinking, which suggested an overall upward developmental trend with some discontinuities, found that performance improvements were moderated by ‘DT test, task content domain, intellectual giftedness, and country of study’ (Said-Metwaly et al., 2020). Downturns are often attributed to children’s improved reasoning abilities, which emphasize convention and logic over creativity and imagination. Other aspects of creativity such as ‘elaboration’ (largely a measure of detail in responses) consistently shows age-related improvement as children have more sophisticated language and greater dexterity (Kim, 2011).

There are now hundreds of lab studies which have sought evidence on the relationship between EC and creativity. It is perhaps predictable that findings present a messy picture given the multi-componential profile of both constructs and the intrinsic differences in the instruments used to study them. Their relationship may be dynamic, altered by task factors, by individual factors such as motivation and personality, and by context (Amabile and Pillemer, 2012; Barr et al., 2015; Pinho et al., 2016; Ivancovsky et al., 2018). Static behavioral tests are not equipped to capture this dynamism and complexity. Mapping test outcomes (measured as products) to processes depends on the ingredients of that process having been comprehensively charted, something which is distinctly not the case for the creative process, particularly in children.

The issue of how to “gain insights into someone’s mental processing as they create…is one of the most intractable problems of creativity research” (Sowden et al., 2020, p. 314). While it is straightforward to measure someone’s ‘original and valuable’ ideas for what to do with a paperclip, when we want to understand *mechanisms* of learning, we are often more interested in *how* they did it. Did ideas come spontaneously? Or were explainable strategies used to seek them out? Do individuals favor one approach or use a mixture? Does the main variation over time occur within individuals, or across individuals? One way to answer these sorts of questions about an individual’s creative process is to ask them.

This is a surprisingly contentious idea. The reluctance to utilize ‘introspective reports’ to examine cognitive processes is partly thanks to a highly influential paper, Nisbett and Wilson’s (1977) ‘Telling more than we can know.’ It reports on a series of experiments in which participants are asked to give explanations about their behavior which has, unbeknownst to them, been covertly manipulated. Nearly all give explanations which do not include the manipulation, leading the authors to conclude that, “There may be little or no direct introspective access to higher order cognitive processes” (Nisbett and Wilson, 1977, p. 231). This work, subsequent replications, and critiques (Shear and Varela, 1999; Johansson et al., 2005, 2006; Petitmengin, 2011; Petitmengin et al., 2013; Fazelpour and Thompson, 2015) will not be considered in detail here (for that see Rogers, 2021). But the important point is that our tendency, when asked to describe a cognitive process, “to slip surreptitiously from the descriptions of our actual experience toward the verbalization of justifications beliefs, explanations, generalizations and abstract knowledge about our experience” (Petitmengin et al., 2013, p. 656) can be avoided. Detailed elicitation interviews using specific prompts, and questions with a strict focus on description not interpretation can guide participants to avoid generalities and stay focused on thoughts situated in a specific time and space.

The bleak verdict on the value of self-report for cognitive insight is not inevitable. Several creativity researchers have highlighted a need to be more open to these approaches (e.g., “Creativity could benefit from greater valuation of subjective self-reports from participants,” Barr, 2018, p. 28). For the current work, the technique of stimulated recall (Bloom, 1953; Calderhead, 1981; Lodge et al., 2000; Morgan, 2007; Després, 2021) is underpinned by the idea that people can vividly relive a situation when they are presented with sufficient cues to the stimuli which characterized the original (Bloom, 1953). The technique often involves the use of audio or video recordings to aid recall (Henderson and Tallman, 2006; Després, 2021). It has been successfully used in children (Lyle, 2003; DeWitt and Osborne, 2010; Määttä and Järvelä, 2013; Meier and Vogt, 2015) though this to our knowledge, is the first example of its use in creativity research in children.

This sort of qualitative research can provide the tools to look at process. It can reflect the unfolding of a thought process over time, in seconds, minutes, or hours. Unlike quantitative creativity studies, where time is often seen as an irksome complicating factor, chronometric qualitative analysis allows us to consider behavior that unfolds in different timeframes; we can consider details within a child’s specific response to a specific question as well as analyze their entire set of responses, to consider whether their approach varied at different times or in different contexts. While quantitative research can help us answer questions about what children did, qualitative can give us insight into how they did it.

We will report on a quantitative study examining the relationship between creativity and EC, a qualitative study examining the relationship in a subset of the same children and the triangulation of findings between the two approaches. The rationale of this ‘mixed methods’ approach (Tashakkori and Teddlie, 2010; Creswell and Clark, 2011) is that combining quantitative and qualitative approaches provides a fuller, more rounded understanding than either method alone (Fetters et al., 2013). In particular, here the quantitative aspect will measure creative product while the qualitative will focus on the creative process. Creativity is especially needful of mixed methods given that producing creative ideas is inherently unpredictable and difficult to replicate. Issues of time constraint, motivation, domain specificity, fragility, and whimsy all threaten the reliability of quantitative lab measurements.

Bringing together quantitative and qualitative data is not an easy task, theoretically or practically (Yardley and Bishop, 2008). Theoretical problems can stem from epistemological incompatibility, while pragmatic problems arise from the sheer complexity of assessing highly diverse data (Willig and Rogers, 2017). An ideal for a fully triangulated design is one in which different but complementary data are obtained on the same topic (Morse, 1991; Johnson et al., 2007) and brought together so that the results, through cross verification from multiple sources (Fielding, 2010; Creswell and Clark, 2011) are both robustly laboratory tested and ecologically valid.

The key questions we address in this work are:

- To what extent are there individual differences in the role EC plays in children’s creativity?
- Does inhibitory control have a detrimental effect on children’s creativity?

## 2. Materials and methods

### 2.1. Ethics statement

The study was given ethical approval by the Departmental Ethics Committee of Birkbeck’s Department of Psychological Sciences, reference 161,744. Safeguarding procedures were carried out in accordance with Birkbeck and Centre for Brain and Cognitive Development policy documents and online advice from the Care Quality Commission and the National Society for the Prevention of Cruelty to Children.

### 2.2. Participants and recruitment

Forty-nine primary school children were tested at university outreach events held over two half term holidays. Children were recruited through flyers distributed to schools, nurseries, and play centers in socioeconomically and ethnically diverse areas of London. Participants spent a half day at the university engaged in group-based, brain-related pedagogical activities, as well as taking part individually in research. Data were collected by a team of researchers.

Four children had incomplete tests and were excluded from the analysis. The final sample was 45 children, aged between 4.95 and 11.36 years (*M* = 7.97; *SD* = 1.82); 24 were girls. The numbers in each age group are shown in Table 1.

**Table 1:** Numbers and ages of participants in quantitative and qualitative studies.

Age (years)	Number in quantitative sample	Number in qualitative sample
4	1	
5	8	
6	7	2
7	7	3
8	7	2
9	7	4
10	7	3
11	1	

Participants for the qualitative study were also from this group. All participating parents were contacted and the qualitative study outlined. Parents then discussed the study with their child and responded on whether they wanted to go ahead. Children were interviewed at home. All children who showed an interest in participating were included in the study. In total, 14 children took part, 4 boys and 10 girls. Their ages are shown in Table 1.

### 2.3. Quantitative study

#### 2.3.1. Power analysis

Power analysis (Cohen, 1992) was carried out using G*power 3.1 (Faul et al., 2007). The main analyses were the correlations between EC and creativity measures, effects without well-established benchmarks of expected size in children. A medium effect size (at 80% power and a probability level of 0.05) of 0.3 correlation would need a sample of 84 for reliable detection. In practice, the number of participants was limited by guidance on safe and enjoyable attendance (i.e., our priority was ensuring the event was fun and relaxed for the children), meaning the study was statistically somewhat under-powered. Results should be considered with that in mind.

#### 2.3.2. Procedure

Children were tested individually in half hour sessions – one each for creativity tests, EC tests and cognitive ability tests. For the verbal tests, children answered out loud with the researcher recording their responses. Between sessions, children participated in semi-structured crafts and games about the brain.

#### 2.3.3. Materials

##### 2.3.3.1. Creativity measures

The Alternative Uses Test (AUT, Guilford et al., 1978) requires participants to generate as many ‘interesting and unusual’ uses for an everyday object. Children were given 3 min to respond. Answers were scored for fluency (total number of responses), flexibility (total number of categories) and originality. Originality was scored by four independent raters, instructed to score each response on a scale of 1 (not creative) to 5 (highly creative). Participant scores were calculated as the mean response originality score (i.e., total originality divided by fluency).

The Torrance Tests of Creative Thinking (TTCT, Torrance, 1974, 2016) are several tests in verbal and figurative domains. The verbal test was ‘Just suppose’, which requires children to initiate creative thought from an imaginary situation (e.g., ‘Imagine clouds have strings attached which come all the way down to the ground’) over 5 min. As with the AUT, fluency, flexibility and originality are scored, according to the TTCT manual. Here, originality is scored according to an in/out system; each response not listed in the manual’s common responses receives 1 point for originality; score per child is the sum.

The only exclusions for fluency were responses which repeated the instruction wording. Inter-rater reliability (IRR) for fluency, calculated on 25% of the sample, was *α* = 1 for AUT and *α* = 0.99 for Just Suppose. IRR for flexibility, defined as a ‘change or shift in attitude or focus’, and originality were also high [*α* = 0.89 for AUT and *α* = 0.69 for JS flexibility; *α* = 1 (JS) and *α* = 0.80 (AUT)] for originality.

The TTCT figural tests are simple paper and pencil drawing games in which children complete drawings around a range of starting stimuli such as circles or parallel lines. Children had 5 min to respond.

The tests were scored according to the TTCT manual instructions, for fluency (number of completed pictures), originality (number of completed pictures not on the list of exclusions), elaboration (number of details added) and overall creative strengths - specific bonus points for signs of creativity in 13 categories (e.g., humor, fantasy, emotional expressiveness). Given the detailed nature of the scoring instructions in the manual, figural tests were scored by just one researcher.

##### 2.3.3.2. EC measures

###### 2.3.3.2.1. Inhibitory control 1: animal size Stroop

**Figure 1:**
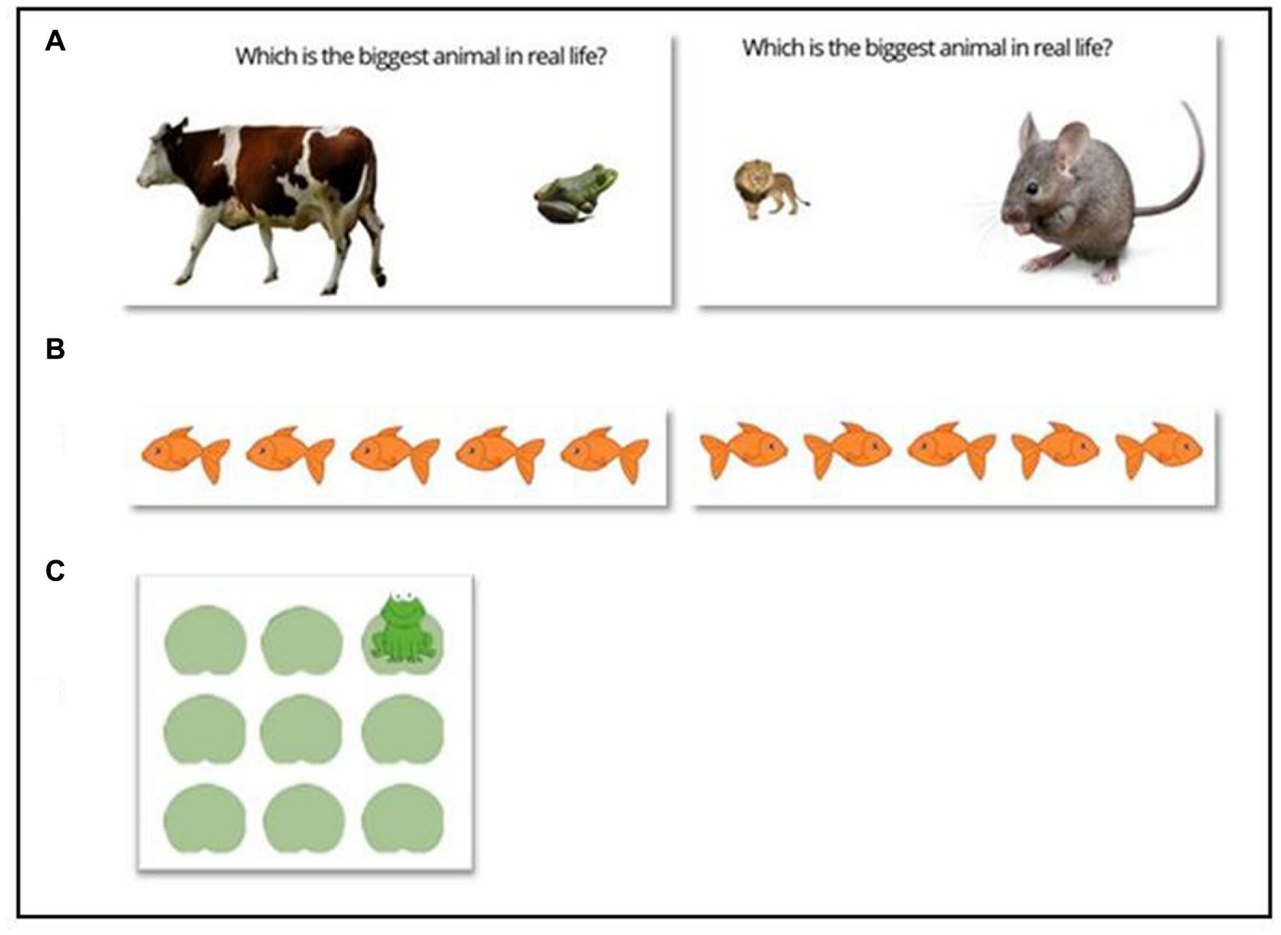
**(A)** Examples of animal size Stroop trials, congruent (left) and incongruent (right). **(B)** Examples of simple Flanker trials, congruent (left) and incongruent (right). **(C)** Example stimulus for visuospatial working memory (VSWM) Corsi block task.

A child-friendly version of Stroop (Stroop, 1935; Catale and Meulemans, 2009; Morris, 2020) programmed in Matlab 9.1.0, was used. The design was based on Merkley et al. (2016). Children were presented with two animal pictures, one large, one small, on a screen (see Figure 1A). They had to decide which animal is larger in real life, irrespective of picture size. This meant inhibiting perceptual characteristics of the stimulus to answer in terms of their knowledge of the animals’ real relative sizes. This inhibition is more difficult (takes longer) when the relative sizes are incongruent (e.g., the lion image is smaller than the ladybird). Children responded by pressing a key on the keyboard on the left or right, corresponding to the large animal. The large animals were lion, horse, cow, and elephant; the small animals mouse, frog, rabbit, and ladybird. Large images were 72 mm x 54 mm; small images 29 mm x 21 mm. Each trial lasted a maximum of 3 s, after which an error was recorded. The inter-trial interval varied between 600 and 1,400 milliseconds (ms) to deter anticipatory or automatic responses. The images were presented in pairs on a screen 50 cm away, in a quiet room. Children were asked to respond as quickly as they could while trying to answer correctly. Reaction times and accuracy were recorded.

###### 2.3.3.2.2. Inhibitory control 2: simple Flanker

The Flanker is a test of selective attention (Eriksen, 1995). In the adult version, respondents press a key corresponding to the direction of a central arrow (Rueda et al., 2004); here the version, based on Anwyl-Irvine A. L. et al. (2020) was programmed in Gorilla^1^ and used brightly colored fish instead of arrows. Children saw a horizontal row of fish in the middle of the screen (see Figure 1B) and had to decide which way the central fish was swimming (left or right), responding with a corresponding left or right key press. Sometimes the central fish is surrounded by fish swimming in the same direction (congruent condition), sometimes the surrounding fish are swimming the opposite way (incongruent condition). Reaction times in congruent trials are quicker than in incongruent trials (Eriksen, 1995; Anwyl-Irvine A. et al., 2020; Massonnié, 2020).

Inter-trial intervals varied randomly between 600 and 1,400 ms. For each trial, a fixation cross was displayed for 1700 ms followed by the screen with fish, which remained on screen until a response was recorded. There were 12 practice trials, with immediate feedback, followed by four blocks of 24 trials each. 50% of trials were congruent, 50% incongruent and trials were randomized for the direction of the central fish. Children were asked to respond as quickly and accurately as possible. Accuracy (proportion of correct trials) and reaction times (RTs) for correct responses were recorded.

###### 2.3.3.2.3. Working memory 1. Verbal working memory

Verbal working memory (VWM) was tested with a backwards digit span task (St Clair-Thompson and Gathercole, 2006). Children faced the researcher and repeated out loud, in reverse order, a list of numerical digits read to them. List length began with two digits, with four trials at each list-length level. Children answering correctly on three or more trials moved on to the next level, where list length increased by one digit. They continued until they failed at two or more trials in a level. The total number of correct trials was recorded.

###### 2.3.3.2.4. Working memory 2. Visuospatial working memory

Children’s visuospatial working memory (VSWM) ability was assessed using a child-friendly, computerized variant (Morris, 2020) of the Corsi block task (Corsi, 1972). Participants were seated 50 cm from a computer screen in a quiet room. They watched a frog make a series of jumps on a 3 × 3 grid of lily-pads (see Figure 1C) They were instructed to click on the lily-pads in reverse order, using the mouse, to indicate the lily pads where the frog had jumped. Children had up to 5 2-jump practice trials to ensure they understood the task, before moving on to test trials. Test trials began with a sequence length of 2, with 4 trials at each level. Children answering at least 3 correctly progressed to the next level, with sequence length increased by 1 jump. They continued until they made 2 or more mistakes at any level. The total number of correct sequences was scored.

##### 2.3.3.3. Tests of cognitive ability

Verbal and non-verbal measures were assessed, according to manual guidelines, to benchmark the sample and correlate test scores with other metrics. The British Picture Vocabulary Scale version 3 (BPVS-III) (Dunn et al., 1997), a test of receptive vocabulary, requires the child to choose which of four pictures corresponds to a word read aloud. The words get progressively more challenging and testing stops when the child makes 8 errors within any block. All children completed the test from the start and raw scores were recorded. BPVS-III norms for children 3–16 have a reliability of 0.91 (Dockrell and Marshall, 2015). Non-verbal abilities were measured using Raven’s progressive matrices (Raven, 2000) which require the child to select from a range of missing elements in an abstract pattern series. The test has split-half reliability of *r* = 0.85 for 5–8 year old children (Carlson and Jensen, 1981).

### 2.4. Qualitative study

The qualitative study took place in participants’ homes, several weeks after the outreach event. Children were told the research was about their creative thinking. It was emphasized that they were the experts, since only they knew what was in their heads. It was emphasized that there were no wrong answers and efforts were taken to ensure children felt comfortable and empowered, through giving them agency in choice of materials, domain and timing.

They chose whether to do a storytelling or drawing activity and were given ‘sparks’ (pictures or shapes) to ensure that their work was novel. They were asked to produce something ‘new and creative’, were allowed to complete their activity with an explicit lack of time constraint and were videotaped doing it. Immediately afterwards, they were interviewed about their work, using the playback of the video, the sparks and their picture or story as stimuli to prompt their recall of their thoughts while they were creating. The audio-recorded interviews were transcribed and analyzed within a theoretically grounded thematic analysis framework.

All children chose their own pseudonyms.

#### 2.4.1. Analytical method

The analysis involved several stages which built up iteratively and recursively (Braun and Clarke, 2006). The first stage involved listening back to interview recordings and transcribing them. Then scripts were parsed line by line twice; first to record everything evoked by each line, the second with a focus on the research question: *How do control processes contribute, positively or negatively, to X’s creativity*? For these, a description starting with the child’s own words (e.g., ‘an idea just popped up’) was recorded for each line, resulting in many descriptive statements per interview. The subsequent analytic stages involved grouping descriptions into themes and a recursive shuttling between descriptions and themes, refining, renaming, and finessing themes and checking the best fit of descriptions within each. This process typically produced between 9 and 15 themes per child. The subsequent stage involved grouping themes into fewer over-arching themes – the primary themes, of which there were between 3 and 5 per child. The final stage involved bringing together themes across the sample of children.

#### 2.4.2. Methodological integrity

Given there remains skepticism regarding the validity of introspective techniques applied to cognition and that this approach with children was novel, we wanted some way of ‘stress testing’ the data. After considering the array of potential threats to veracity of the data, we developed a novel checklist, ‘The 7 Cs,’ to help assess data validity. The checklist, alongside the recordings, would also make it theoretically possible for an external evaluator to assess the validity of children’s responses. The checklist involved asking whether each child’s data was sound in terms of:

- Cooperation. Was the child willingly and happily involved?
- Consistency. Did the child’s account at different times match up?
- Confirmation. Did their accounts concur with secondary evidence (e.g., the video evidence demonstrating the order in which events occurred)?
- Corroboration. Did the child’s account corroborate well-evidenced descriptions in the creativity literature (e.g., functional fixity)?
- Contradiction. Did the child correct the experimenter’s version of their account, thereby showing a greater degree of certainty of their own?
- Coherence. Did the child’s responses make sense?
- Confidence. Did the child articulate their thoughts with certainty?

The purpose of thematic analysis is to identify and understand recurrent patterns which occur across data sets – within an individual and between them. Here, the units of analysis were generally phrases, though sometimes whole sentences and even whole transcripts were considered, since this analytical method allows ‘zooming out’ as well as ‘zooming in’ (Braun and Clarke, 2006). For example, an account might, in one part of the interview, describe high involvement of control processes during planning, but then, in another part of the interview, describe low involvement of control, for example while telling the story or drawing. In such instances, descriptions might emerge not from the child’s words *per se*, but from a comparison of their accounts at different times. In this way the dynamic nature of the relationship between control processes and creativity could be captured.

## 3. Results

### 3.1. Quantitative study results

We will first present findings on general cognitive ability data, to benchmark the sample against norm scores and established developmental trajectories. We will then present EC and DT test results separately before considering the relationship between them.

#### 3.1.1. General cognitive ability

Descriptive statistics for scores on the BPVS and Ravens are shown in Table 2. Expected norm scores and their ranges for both tests are given on the basis of our sample’s mean age of 7.97 years.

**Table 2:** Means, SDs and range of scores on BPVS and Ravens (raw scores) and norm equivalents.

	Mean raw score	Norm score mean (75% CIs)	SD	Min	Max
BPVS	114.44	108 (85, 115)	26.03	29	157
Ravens	27.63	25 (17, 33)	6.16	12	34

Simple regression analyses (Table 3) tested whether, as expected, age predicted outcomes on each measure; results showed it did for both.

**Table 3:** Simple regression analyses of age on each measure of cognitive ability.

	*B*	SE(b)	*β*	*F*(1,44)	*p*	*R* ^2^
BPVS	10.54 (7.52, 13.55)	1.49	0.74	49.75	<0.001	0.55
Raven’s	2.40 (1.65, 3.14)	3.04	0.71	42.36	<0.001	0.51

#### 3.1.2. Executive control

##### 3.1.2.1. Working memory

Results for the two measures of working memory are shown in Table 4. Correlation between the two measures was significant and moderately high: Pearson’s *r* = 0.41, *p* < 0.001.

**Table 4:** Means, SDs and range of scores for visuospatial and verbal working memory (number correct), Stroop and Flanker reaction time difference (milliseconds), and creativity tests Alternative Uses Test (AUT), Just Suppose (JS) and Figural (scores).

	Mean	SD	Min	Max
VSWM	7.32	4.39	0	15
VWM	8.24	2.85	0	13
Stroop RT diff	75	90	−136	290
Flanker RT diff	43	79	−50	352
AUT fluency	8.61	4.32	2	21
AUT flexibility	6.87	3.46	0	17
AUT originality	2.72	0.63	1	4
JS fluency	11.13	6.37	1	34
JS flexibility	4.17	3.27	0	16
JS originality	8.61	5.72	0	29
Figural fluency	5.48	1.68	2	12
Figural originality	4.28	1.87	1	11
Figural Elaboration	5.43	2.33	2	11
Figural creative strength	2.78	2.19	0	9

##### 3.1.2.2. Inhibitory control

###### 3.1.2.2.1. Pre-processing of inhibitory control measures

Accuracy was at ceiling (>92%) for both Flanker and Stroop so reaction times (RTs) for correct answers were used as the main measure. RTs shorter than 200 ms were excluded as probable anticipatory reactions (Anwyl-Irvine A. et al. 2020). RTs more than three standard deviations from the mean for each participant were excluded so extreme values did not affect results (Anwyl-Irvine A. L. et al. 2020). The traditional measure of ‘Stroop cost’ and ‘Flanker effect’ i.e., difference in RT between incongruent and congruent trials, was used for analysis.

Analyses of variance, with congruency as a within-subject factor, were carried out. For Flanker, RTs were significantly longer for incongruent (*M* = 941 ms) than congruent (*M* = 897 ms) trials [*F*(1, 40) = 12.36, *p* = 0.001, η^2^*_p_* = 0.24]. For Stroop, RTs were also longer for incongruent (*M* = 1,048 ms) than congruent (*M* = 968 ms) trials [*F*(1, 40) = 38.45, *p* < 0.001, η^2^*_p_* = 0.50].

For each participant, an RT cost score was calculated as the mean RT for correct incongruent trials minus the mean RT for correct congruent trials. Higher scores thus represent poorer inhibitory control. Results are shown in Table 4.

### 3.2. Creativity test results

#### 3.2.1. AUT

All children produced at least two ideas for alternative uses for everyday objects. There was wide variation in fluency and flexibility. Originality scores were constrained by the method of scoring. An example answer scoring highly for originality (for pencil) was ‘use the lead to poison my sister’; a low scoring example was ‘poke a hole with it’. Results are shown in Table 4.

#### 3.2.2. Just suppose

Some children produced just one idea in the imaginary scenario and there was again wide variation in scores, as shown in Table 4.

#### 3.2.3. Figural tests

All children completed two out of three possible figural tests, with each scored for fluency, originality, and elaboration. Total scores were simple sums from the two tests. In addition, their total figural output (i.e., all drawings together) was assessed for creative strengths, according to the Torrance guidelines, to produce a ‘creative strength’ score. Results are shown in Table 4.

#### 3.2.4. Correlations within constructs

##### 3.2.4.1. EC

Pearson’s correlations were used to look at the relationship within and between measures of the two EC factors. Results (Table 5) showed that tests within the same factor showed significant correlations while those between factors did not. The negative direction of correlations between WM and IC measures is to be expected given high WM and low IC scores represent better performance.

**Table 5:** Pearson’s correlations (top), and significance levels (bottom), for EC measures.

	VSWM	VWM	Stroop	Flanker
VSWM		0.41*	−0.15	−0.24
		0.012	0.375	0.162
VWM			−0.26	−0.24
			0.089	0.126
Stroop RT diff				0.31*
				0.05

In all tables *n* = 46, correlations, not corrected for multiple comparisons, are marked with * are significant at the *p* < 0.05 level, correlations marked.

##### 3.2.4.2. Creativity

Pearson’s correlations were similarly used to look at the different tests and dimensions of creativity tests. As with any case involving exploratory study of multiple comparisons, caution is advisable in interpreting findings. The verbal tests were highly correlated in the constructs of fluency and flexibility while originality scores were not. In correlations between verbal and figural domains, the picture was mixed. Some sub measures were highly correlated across domains (e.g., figural elaboration with most verbal measures) while others appeared distinct; neither fluency nor originality in the figural domain was significantly related to its counterpart - or indeed any other sub measure - in the verbal tests. Full results are shown in Table 6.

**Table 6:** Pearson’s correlations (top) and corresponding significance levels (bottom) between sub measures across verbal and figural domains.

	Verbal tests				
	AUT fluency	AUT originality	JS fluency	JS flexibility	JS originality
Figural fluency	0.11	−0.01	0.18	0.18	0.13
	0.472	0.972	0.235	0.225	0.387
Figural originality	−0.03	−0.08	0.12	−0.02	0.08
	0.872	0.606	0.427	0.919	0.601
Figural elaboration	0.24	0.41**	0.29	0.31*	0.26
	0.109	<0.001	0.051	0.036	0.078
Figural creative strength	0.36*	0.11	0.30*	0.29*	0.28
	0.014	0.472	0.045	0.047	0.057
AUT fluency			0.57**	0.66**	0.59**
			<0.001	<0.001	<0.001
AUT flexibility			0.66**	0.75**	0.66**
			<0.001	<0.001	<0.001
AUT originality			0.26	0.22	0.25
			0.08	0.15	0.09

In all tables *n* = 46, correlations, not corrected for multiple comparisons, are marked with * are significant at the *p* < 0.05 level, correlations marked. with ** are significant at the *p* < 0.01 level.

#### 3.2.5. Relationship between EC and creativity measures

The final step considered the relationship between each EC and creativity variable, controlling for age. Results are in Table 7. Looking first at working memory, AUT originality showed significant positive correlation with VWM but not VSWM. JS measures were inconsistently correlated. The figural test sub measures showed no significant correlations with WM except figural originality which was inversely correlated with VWM. For inhibitory control, the general pattern was a lack of significant correlations between both inhibitory control measures and all creativity sub measures, both verbal and figural. The only exception was a significant moderate correlation between Flanker and figural originality.

**Table 7:** Pearson’s *r* (top) and significant values (bottom) between EC and creativity measures.

	AUT			JS			Figural			
	flu	flex	orig	flu	flex	orig	flu	orig	elab	strngth
VSWM	0.16	0.11	0.23	0.3	0.06	0.28	−0.06	0.1	0.05	0.29
	0.359	0.514	0.174	0.078	0.73	0.097	0.693	0.566	0.787	0.091
VWM	−0.12	−0.1	0.36*	0.15	0.1	0.11	−0.06	−0.42*	−0.19	−0.32
	0.488	0.558	0.03	0.376	0.572	0.532	0.706	0.011	0.279	0.058
Flanker	−0.22	−0.16	−0.26	−0.05	−0.02	−0.01	0.04	0.33*	0.07	0.03
	0.177	0.309	0.096	0.781	0.904	0.972	0.813	0.036	666	0.875
Stroop	0.12	0.1	0.14	0.03	−0.05	−0.03	0.11	0.23	0.07	0
	0.451	0.522	0.395	0.838	0.762	0.849	0.514	0.157	0.687	0.994

In all tables *n* = 46, correlations, not corrected for multiple comparisons, are marked with * are significant at the *p* < 0.05 level, correlations marked. with ** are significant at the *p* < 0.01 level.

#### 3.2.6. Summary

Correlations between creativity and EC measures presented a mixed picture; VWM showed significant correlation with two originality measures, one verbal (AUT) and one figural, while VSWM showed no significant correlations. For IC, the only significant correlation was between the Flanker and originality in the figural domain. The results did not give a clear answer of the extent to which there are individual differences in the role EC plays in children’s creativity; the lack of significant correlation could be due to a high level of individual variability but could also be due to the study being underpowered. If the explanation lies in a high level of individual variability, it is worth noting that even a more highly powered study might fail to detect it. On the question of whether inhibitory control has a detrimental effect on creativity, the correlational evidence suggested that it did not.

### 3.3. Qualitative study results

The analysis distinguished three primary themes, described below. Descriptions and illustrations of sub-themes follows.

1. Descriptions of thought which appear spontaneous, free-wheeling, and uncontrolled. Ideas, refinements, or adaptations arise without deliberate effort, from the senses, from memory or from new spontaneous associations.
2. Descriptions of thought which appear controlled or focused. These are ideas being deliberately elaborated, planned out, evaluated, adapted, or even formed anew in a deliberate, strategic way.
3. Descriptions of processes concerned with adjusting the balance between spontaneous and controlled processes. Managing and adapting constraints and switching between spontaneous and controlled processes either spontaneously or deliberately. This category also includes descriptions of failure at the extremes, specifically, excess control leading to a lack of ideas or overly strict censorship of them, and excess spontaneity leading to an overload of ideas and difficulty selecting from them.

**Figure 2:**
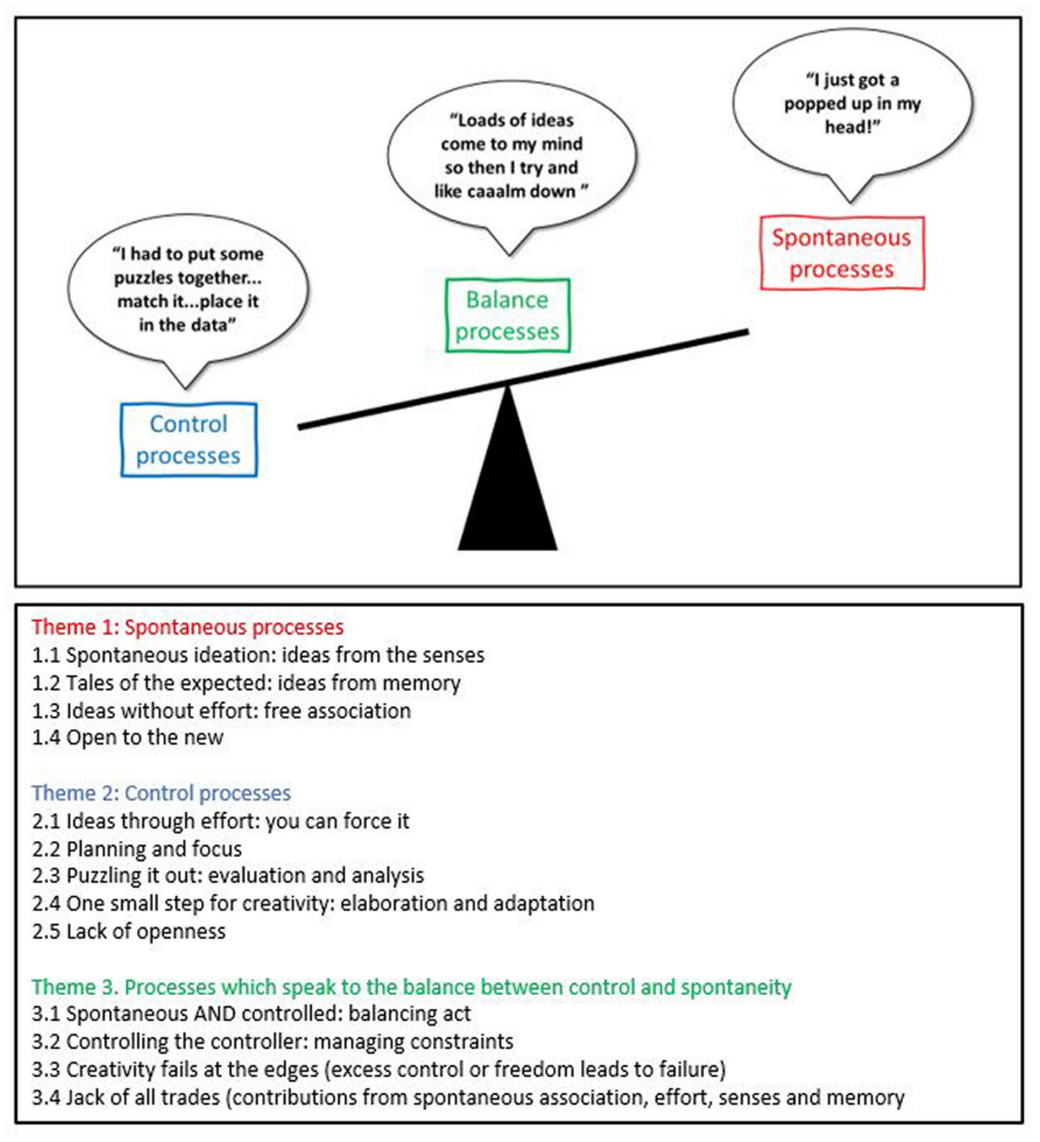
The three primary themes of creative thought, with illustrative quotes and (below) full list of primary and secondary themes.

These themes are illustrated in Figure 2 below, to show the relationship between them. The relationship is shown as a balance beam, with spontaneous processes at one end, control at the other and balance processes as the means by which the beam is moved. The sub themes are also shown in the lower part of Figure 2.

In the following section, a description of each sub theme is given with illustrative quotes from participants (names are pseudonyms). These derive from narratives as the children watched back videos of themselves some minutes after creating; they were not contemporaneous with the creative process, since this might have interfered with the process.

#### 3.3.1. Primary theme 1. Spontaneous processes

##### 3.3.1.1. Spontaneous ideation: ideas from the senses

For some children, spontaneous ideation was primarily driven by perceptual experience in the immediate environment.

Dave*, ‘When I look outside there are there’s birds in the sky, so I think I’ll draw a few birds.’*

##### 3.3.1.2. Tales of the expected: ideas from memory

Children also described ideas coming from memory sources, including life experiences and memories from fiction.

Betty*, ‘I might use the same ideas from the movie… I just change a few things’.*

Imagination Creation, [Ideas come..] *‘from things you do, read, watch, anything like that*’.

##### 3.3.1.3. Ideas without effort: free association

Spontaneously making new associations, connecting elements from memory, from senses or both, was another commonly described spontaneous process.

Snowy, ‘*The blackberry popped in from the TV and this abacus here - because the TV is how black the blackberry was and one of these balls is how small the blackberry was.*’

Imagination Creation, ‘*I was actually like thinking I like superheroes and I like the one that could shape shift … I was going to do broccoli but if I did corn that could turn into popcorn so that was enough so yeah … .if it’s corn then it’s popcorn so it’s kind of like a real shape shift.*’

##### 3.3.1.4. Open to the new

The final catalyst for spontaneous creative expression was more a trait-like theme of openness. Several children mentioned liking things which were new, unusual, even strange; these might arise from senses, memory or freshly made chance associations.

Dooda, ‘*I’ve drawn a lot of rabbits and I know I like rabbits and I want one as a pet and I like them but I want something new and different.*’

Harriet, ‘*I just like strange things … cos they are interesting.*’

#### 3.3.2. Primary theme 2. Control processes

##### 3.3.2.1. Ideas through effort: you can force it

There were several accounts suggesting ideas can emerge not through spontaneous ‘popping up’ but through deliberate effort, for example, by exploring broad categories or dismantling or rearranging parts of a fledgling idea.

Dooda, ‘*I’m trying to think of a shape that it’s in, and what it’s got on to like for ears … I’m thinking of all the animals that have small ears.*’

Roxy, ‘*I just try and like… maybe swap parts of it or look at it from a different angle or step back and see if I can see something else.*’

##### 3.3.2.2. Planning and focus

Nearly all children were very focused on producing their creative work, concentrating for 30–55 min without a break. Several talked of the need to plan ahead.

Silky, ‘*I was focused on my picture most of the time… When I was drawing my dock erm and I had not drawn fishes I decided to add fishes but finish what I was doing first.*’

Betty, ‘*I knew I had to go back over things so I thought to just do it at the end.*’

##### 3.3.2.3. Puzzling it out: evaluation and analysis

In describing how to evaluate candidate ideas for selection, children sometimes brought reality into their imaginary scenarios to judge ideas.

Lexy, ‘*A house could be in the background but who would want to live behind a Viking ship with Vikings shooting cannons? No one.*’

Dave, ‘*Do pigs live in sandy places? No. Would a witch want to be in a desert to do a spell? I do not think so.*’

##### 3.3.2.4. One small step for creativity: elaboration and adaptation

Control processes were widely in evidence in the small elaborations, adaptations and refinements that are customarily part of the creative process. The elaboration phase is often a time for ‘taking a step back’ and looking at work with new eyes.

Ben Ten, ‘*I made this bit a bit shorter because last time it went too long and I curved more because last time I went straighter.*’

Harriet, ‘*Well first I thought I’d do it like the tree trunk is cut in half so you could see the steps but then I decided that was too difficult so I just did a normal tree trunk.*’

##### 3.3.2.5. Lack of openness

Lack of openness manifested at many levels – from the sort of cognitive fixity widely recognised in design research (Jansson and Smith, 1991; Bartholomew and Ruesch, 2018) through to the social inhibition which blocks ideas that might appear silly, wrong, or just plain weird.

Alex, ‘*No I, I like, no I do not like things that are too odd.*’

Lexy, ‘*I do not want to draw a chicken bone cos that’s weird.*’

Dave, ‘*I knew that I wasn’t going to use that one because that does not make any sense at all.’*

#### 3.3.3. Primary theme 3. Balancing control and spontaneity

The third component of processes concerned those modulating the balance between spontaneity and control. This was sometimes deliberate, sometimes (in response to distraction or accident) spontaneous. For some it was determined by a change of domain, for others by a change in the degree of freedom or constraint (for example, applying a rule instigating a shift to greater control). The stage of the creative process was also a factor, with earlier more generative stages typically being freer and more open, and later ones more rigorously controlled.

##### 3.3.3.1. Spontaneous and controlled: balancing act

Several children described a quite deliberate shift between a controlled and a spontaneous thought process, pointing to sophisticated metacognitive awareness:

Betty, ‘*I normally just concentrate… then I realize I’m concentrating too hard and then that’s when normally when my mind goes blank and then I sit back and then I relax and then after it comes back to me… so it’s like my mind is telling me to stop working and then when I relax it comes, the idea comes back to me…*’.

For Roxy, the shift was in response to the stage of the creative process, as her freewheeling approach in the generation phase was followed by a cooler controlled approach for evaluation:

Roxy, ‘*If I was thinking of a subject immediately like loads of ideas come to my mind so then I try and like ‘Caaaaalm down’ and just find one that really captures me*’.

##### 3.3.3.2. Controlling the controller: managing constraints

Many sources, internal and external, led to a temporary or permanent change of constraint.

Alex, ‘*If … like a bird pops into my head but that does not really look like a wing I could say erm it was like going to be a mythical bird or something*’.

Dooda, ‘*When I thought of Dover castle it did not really have bricks but I thought that it does not matter I’m not drawing Dover castle*’.

##### 3.3.3.3. Creativity fails at the edges (excess control or excess freedom lead to failure)

There is risk of failure at both extremes of control and spontaneity, each with distinct implications for creativity. Excess control limits the free flow of ideas, reducing quantity and thereby the chances of surprising, novel associations and ideas. Excess freedom produces sufficient quantity and diversity of ideas but insufficient control could impede effective selection from them.

Excess control often meant a head empty of ideas:

Alex, ‘*I just could not think of anything … I thought like the sun but that’s a bit boring*’.

Betty, ‘*When I’m concentrating too hard … that’s normally when my mind goes blank and … my mind went blank a few times.’*

By contrast, too little control evoked a head full of ideas:

Roxy, ‘*They were all zooming around and I was just yeah maybe I was a bit indecisive*’.

Dooda, ‘*When you think of lots of things well my head just start hurting and I just had a headache.*’

##### 3.3.3.4. Jack of all trades (spontaneous association, effort, senses, memory)

The shuttling between freedom and control described by the ‘Jack of all trades’ theme describes children who, over the course of creating, deployed diverse approaches to creative thinking, some controlled, some spontaneous. Dave described a subtle shift between freer, associative, and controlled, logical thought processes:

Dave, ‘*I chose the sun because it looks exactly like a sun … I started to have an idea that it would be really hot … that’s why I was drawing the sweat on the camel.*’

[What happened here when you drew a smiley face on the camel and then rubbed it out?]

Dave, ‘*I changed it to a huffh [making panting face] and that was because I thought it would be really hot and would you have a smiley face if you were really hot? I do not think so’*

#### 3.3.4. Summary

On our research questions, the qualitative study gave us rich information on the high level of individual variation in how children deployed EC in their creativity. The second research question (whether inhibitory control has a detrimental effect on creativity) cannot be fully answered solely using data from the current study, since we have no direct measure of children’s level of creativity; their creative works were not comparable. There are clues that inhibitory control had detrimental effects in some instances, e.g., as described by the theme ‘Creativity fails at the edges’, but this theme also included reports of failures caused by excessive spontaneity. To get better traction on the question of the effect of inhibitory control on performance, we have to try something else: bring together the current study’s accounts of process with product data from the quantitative tests. We will now discuss the theory and process of data triangulation.

## 4. Triangulation

### 4.1. A word on epistemology

Debates about mixed methods approaches have raged more or less stormily for two decades (Tashakkori and Teddlie, 2009; Tashakkori and Teddlie, 2010; Creswell and Clark, 2011; Hesse-Biber and Johnson, 2015). There is not space here to fully explore these ‘paradigm wars’ (Tashakkori et al., 1998; Teddlie and Tashakkori, 2008) but we do need to acknowledge we are stepping into something of an epistemological no-man’s land. Snipers are picking off weaknesses in validity, robustness, generalizability, and replicability on one side and shortcomings in richness, meaning, detail and depth on the other.

The pragmatic approach taken by many MM researchers (Creswell and Clark, 2011) treads carefully through the minefield, with a focus on “the primary importance of the questions asked rather than the methods” (Creswell and Clark, quoted in Hesse-Biber, 2015). The flexibility of pragmatists to countenance research from multiple perspectives, using all available tools has led some to caution of ‘interdisciplinary opportunism’ (Patai and Koertge, 1994), suggesting that researchers who dabble in new fields do so in a random or uncritical way. In many ways, this is unfair. Consideration of the epistemic basis of data should be a component of any study, not only those which seek to combine data. Nonetheless, pragmatic approaches demand further explanation. The pragmatism underlying this study is based on Morgan (2014) approach which moves “beyond dualistic thinking (e.g., deduction vs. induction, subjectivity vs. objectivity, idiographic vs. nomothetic conclusions) toward more practical choices” (Morgan, 2014, p. 70). Our pragmatism is characterized by critical flexibility, by the view that positions represented as binaries are better seen as ends of continua, and by a dynamism in viewing the research endeavor as an ongoing, recursive, and communicated process. Abductive reasoning shuttles between induction and deduction, as theories are tested by gathering data which in turn inform new theories and so on. The usual forced dichotomy between subjective and objective is similarly replaced with the notion of ‘intersubjectivity’ which is happy to assert “both that there is a single ‘real world’ and that all individuals have their own unique interpretations of it” (Morgan, 2014); and finally, any absolute distinction between the specific and the universal is rejected, in favor of a critical approach which interrogates the extent to which any finding can be applied to other settings and circumstances (Morgan, 1998; Morgan, 2014; Schwandt and Lichty, 2015; Yardley and Bishop, 2008).

While the current research is theoretically grounded in the sort of pragmatic approach Morgan outlines, this still leaves open questions about the logistics and practicalities of bringing together disparate data.

### 4.2. Triangulation methodology

The first step in triangulation involves asking some key reflexive questions: what do these data tell me and crucially, not tell me about? What is the strength of, and how convincing is the claim? How can I make best sense of different forms of data in a way that is consistent with these previous questions? (Mason, 2017). The first questions have been dealt with in previous sections so the focus here is on the third. There are various possibilities:

- Following up similar themes in the different data sets (e.g., comparing whether children with low inhibitory control in quantitative lab tests also give accounts in their qualitative interviews which suggest low levels of control).
- Generating testable propositions and asking them of different data sets (e.g., if the quantitative data show a child has high fluency coupled with high inhibitory control, then the qualitative data could be used to look at how they are achieving this difficult balance according to their own report. Or the reverse: does a child who qualitatively describes a very spontaneous approach show evidence of high levels of fluency in quantitative lab tests?)
- Using different data sources to address a topic from different angles (e.g., at a higher level of description, do children who are at extremes of the control/spontaneity continuum present distinguishable profiles of scores in lab tests of EC and creativity?)

In the full triangulation study (see Rogers, 2021) several attempts were made at triangulation using different approaches. This is not unusual since every case of bringing quantitative and qualitative data together is unique (Mertens and Hesse-Biber, 2012). Here, these false starts will not be reported; only the final approach and the justification for it, will be presented.

#### 4.2.1. Outlining our research questions

The qualitative findings showed that children varied greatly in the quantity and quality of EC involvement in their creativity; also, that there were trait and state differences in their flexibility to shift between more and less controlled approaches. What those data alone could not tell us was whether there was any relation between the approach taken and the creative level achieved – bluntly: were there better or worse ways of ‘doing creativity’? This is the first broad question that the triangulation attempted to address.

The model derived from the qualitative study proposes that there could be creative failure at extremes. Was there evidence of this from our quantitative results? i.e., did children at extremes of ‘spontaneity’ or ‘control’ as evidenced by qualitative work show failures, as proposed, in value and originality respectively?

In essence then, we considered first, whether there was a correlation between children’s use of control, spontaneity, and flexibility (as qualitatively described) and their quantitative results in lab tests of EC and creativity and second, whether there was evidence of creative failure, as measured in quantitative tests of creativity, at extremes of the control/spontaneity qualitative axis?

The data available to address these questions were:

Quantitative data:

- EC scores: Flanker, Stroop, visual and visuospatial working memory.
- Creativity scores: AUT, Just Suppose, TTCT Figural test scores.

Qualitative data:

- Individual level thematic analyses of interview data.

The data on the 14 children who had complete data sets for both quantitative and qualitative tests were prepared in order to address these questions. The first step was to rank each child on each of the three qualitative dimensions: control, spontaneity, and flexibility. This meant going back to the individual child’s thematic analysis and assigning them a score (on a 1 to 10 scale) for each of these dimensions, on the basis of the importance of each in their account. For example, while all children had some examples of spontaneous processes – ideas ‘popping up’ – for some this was a rarity, whilst for others it was pervasive. Flexibility was hardest to rate, since the ways in which children were flexible were varied, e.g., Imagination Creation was flexible moment-to-moment, whilst Betty was flexible over minutes and hours; Alex was more flexible in one domain than another. Scoring children in this way also raised conceptual problems. For example, is the more controlled child the one who notices distractions but articulates the fact they have blocked them out? Or the one who does not appear to notice them?

The quantitative data were also prepared. To control for age differences, standardized residuals from age-predicted linear regression analyses were calculated for each EC and creativity variable. Although some sub measures did not show age-related change, the most consistent approach was to control for age for all variables.

The next step was to enter all the rank scores from the qualitative results alongside the standardized residual scores for each EC and creativity variable and carry out Spearman’s correlations (Table 8). The qualitative data are not being used to ‘predict’ or ‘test’ findings against the quantitative data, but rather both types of data are being considered on a par, with a simple question being asked about their correlation. This represents the effort to avoid a mixed methods approach in which quantitative data dominate by default.

**Table 8:** Spearman’s correlations (top) and corresponding *p* levels (bottom) between qualitative derived rank scores and EC test scores, *n* = 14.

	Flanker	Stroop	VWM	VSWM
Spontaneity	0.36	−0.04	0.02	−0.05
	0.222	0.892	0.961	0.866
Control	−0.08	0.37	−0.46	0.1
	0.795	0.212	0.159	0.738
Flexibility	0.41	−0.1	−0.36	−0.2
	0.163	0.748	0.275	0.517

Despite expectations of associations between the qualitative ‘control’ dimension and measures of inhibitory control in the lab, no significant relationships with lab EC measures were found.

Finding no correlation between qualitative and quantitative measures carries a range of possible meanings: that there really is no relationship between quant-measured lab EC and qual-measured real-world EC, that one or other measure is too noisy, or invalid, or that they are measuring different things. With the smaller numbers typically seen in qualitative (and therefore triangulation) studies, the correlation size needed for a typical significance level of *p* < 0.5 is much higher, so null findings might also be due to sample size.

In the next step, the qualitative rankings were compared with the standardized residual scores on the three creativity tests (Table 9). The results showed that spontaneity was significantly and highly correlated with scores for fluency and flexibility (but not originality) on the AUT, but not with the other creativity tests – surprisingly given the high correlation between AUT and JS sub measures. Control rankings, by contrast, were negatively correlated with fluency and originality (but not flexibility) in the Just Suppose test. Correlations between control rankings and AUT fluency and flexibility were also negative and close to a significance threshold of 0.05 (0.067 and 0.051 respectively). It is notable that out of 9 ‘control’ correlations, 7 were negative. The probability of this number of negative correlations happening by chance, under a simple binomial distribution, is 0.07. Flexibility rankings showed a similar pattern to spontaneity, with positive correlations with fluency and flexibility on the AUT and no other significant correlations. Only the verbal tests showed any significant correlations; the figural tests showed no correlation with any of the qualitatively derived dimensions, suggesting that these tests could be tapping different processes.

**Table 9:** Spearman’s correlations (top) and corresponding *p* levels (bottom) between qualitatively derived rank scores and creativity sub scores, *n* = 13.

	Just Suppose			AUT			Figural tests		
	Flu	Flex	Orig	Flu	Flex	Orig	Flu	Orig	Strength
Spontaneity	0.29	0.4	0.28	0.77**	0.75**	0.18	0.32	0.37	0.53
	0.332	0.176	0.353	0.002	0.003	0.55	0.284	0.213	0.065
Control	−0.64*	−0.49	−0.60*	−0.52	−0.55	0.02	−0.23	0.08	−0.47
	0.017	0.086	0.032	0.067	0.051	0.946	0.46	0.792	0.103
Flexibility	0.15	0.25	0.15	0.67*	0.59*	−0.07	0.21	0.28	0.3
	0.626	0.419	0.635	0.013	0.032	0.814	0.495	0.364	0.329

In all tables *n* = 46, correlations, not corrected for multiple comparisons, are marked with * are significant at the *p* < 0.05 level, correlations marked. with ** are significant at the *p* < 0.01 level.

The tables included here involve multiple comparisons and with the small participant numbers involved, no attempt was made to correct for these multiple comparisons. This should be borne in mind when interpreting the reported significance.

**Figure 3:**
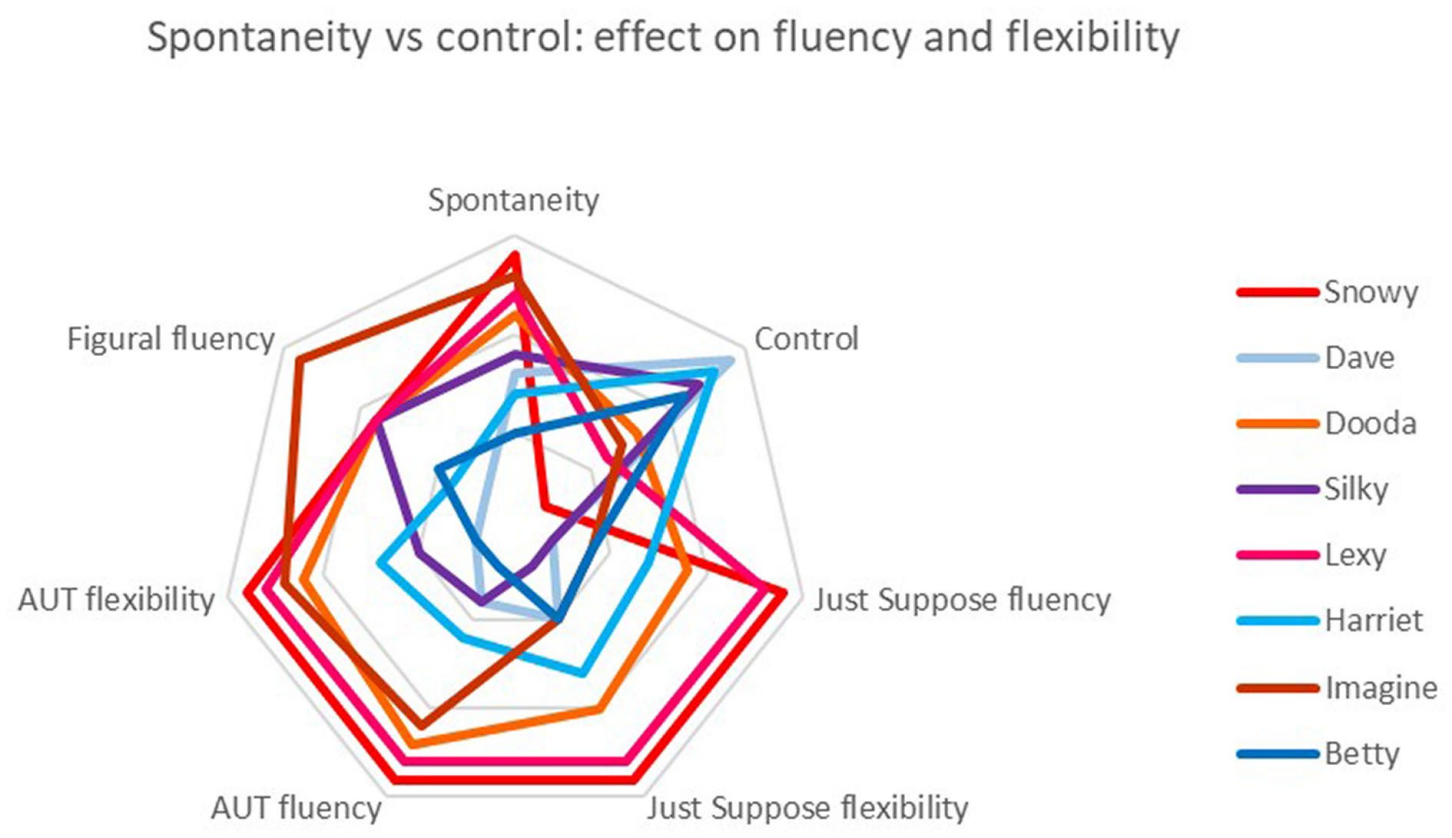
Comparison of the four children ranked highest for spontaneity (red spectrum colors) with the four ranked highest for control (blue spectrum colors) on measures of fluency and flexibility across verbal and figural tests. Note that ‘Spontaneity’ and ‘Control’ are qualitative rankings while the others are quantitative.

**Figure 4:**
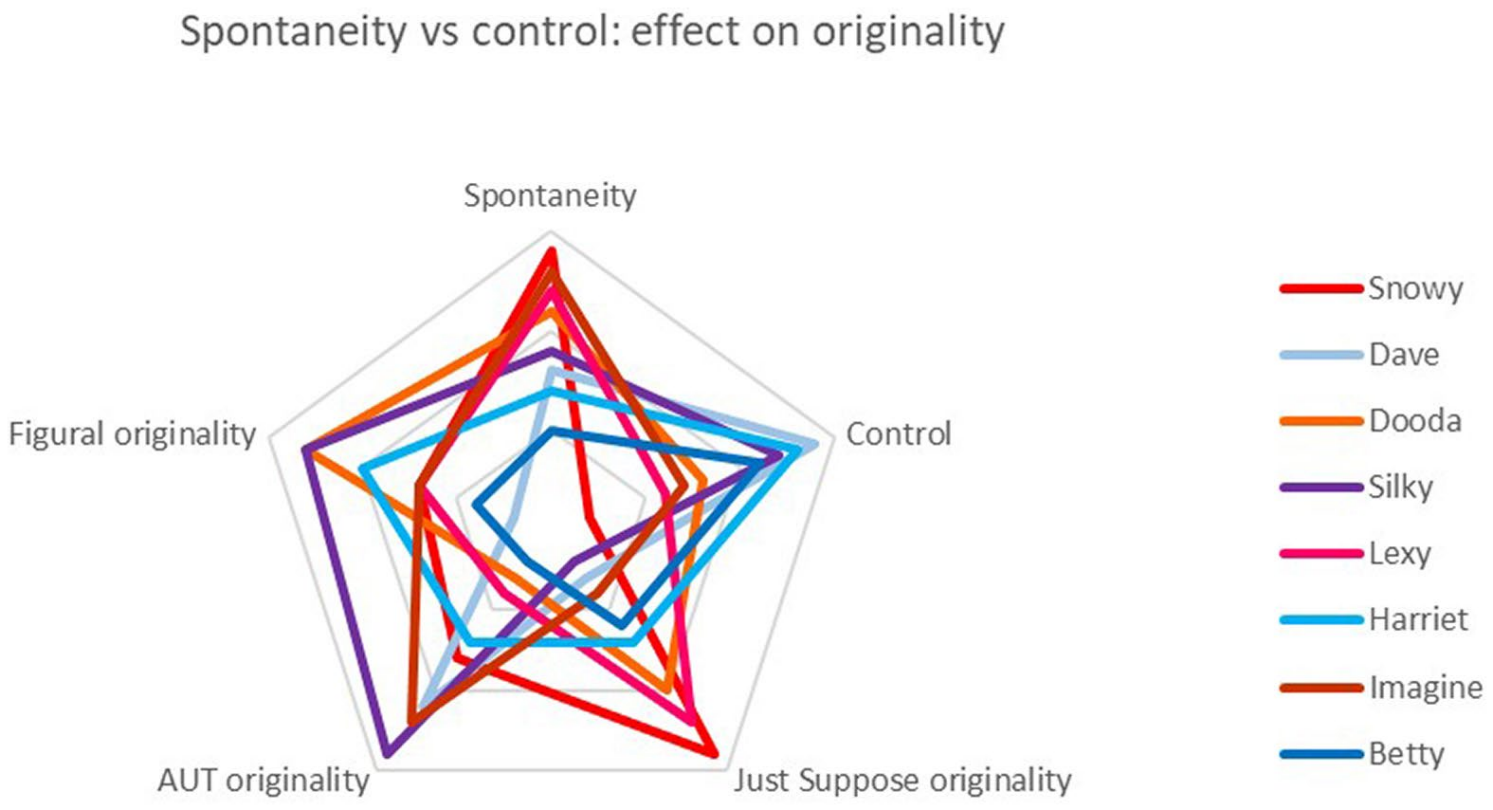
Comparison of the four children ranked highest for spontaneity (red spectrum colors) with the four ranked highest for control (blue spectrum colors) on measures of originality across verbal and figural tests. Again, note ‘Spontaneity’ and ‘Control’ are qualitatively derived, while the others are quantitative.

To address the question of potential failure at extremes of spontaneity or control, rank scores were used for both qualitative and quantitative data. This allowed questions of relative performance to be asked, e.g., Were the children who were most spontaneous also the most fluent? Were those who were most controlled also the least flexible? The analysis compares the four children ranked highest for spontaneity with the four ranked highest for control in the qualitative analysis. The predictions are that the most spontaneous children will be amongst those scoring highest for fluency, flexibility and originality in the quantitative tests, while those ranking highest for control in the qualitative analysis will be amongst the lowest scorers on these variables. Results are presented visually (Figures 3, 4), with red spectrum colors depicting highly spontaneous and blue spectrum highly controlled children. The results show that, for fluency and flexibility, all four ‘spontaneous children’ outperform all four ‘control children’ in all these measures across all tests, verbal and figural.

Turning to a similar comparison with originality scores, the results are more mixed. In the Just Suppose test, the same pattern was seen as with fluency, i.e., spontaneous children outdid control children. But in AUT and figural originality, there was no clear delineation.

**Figure 5:**
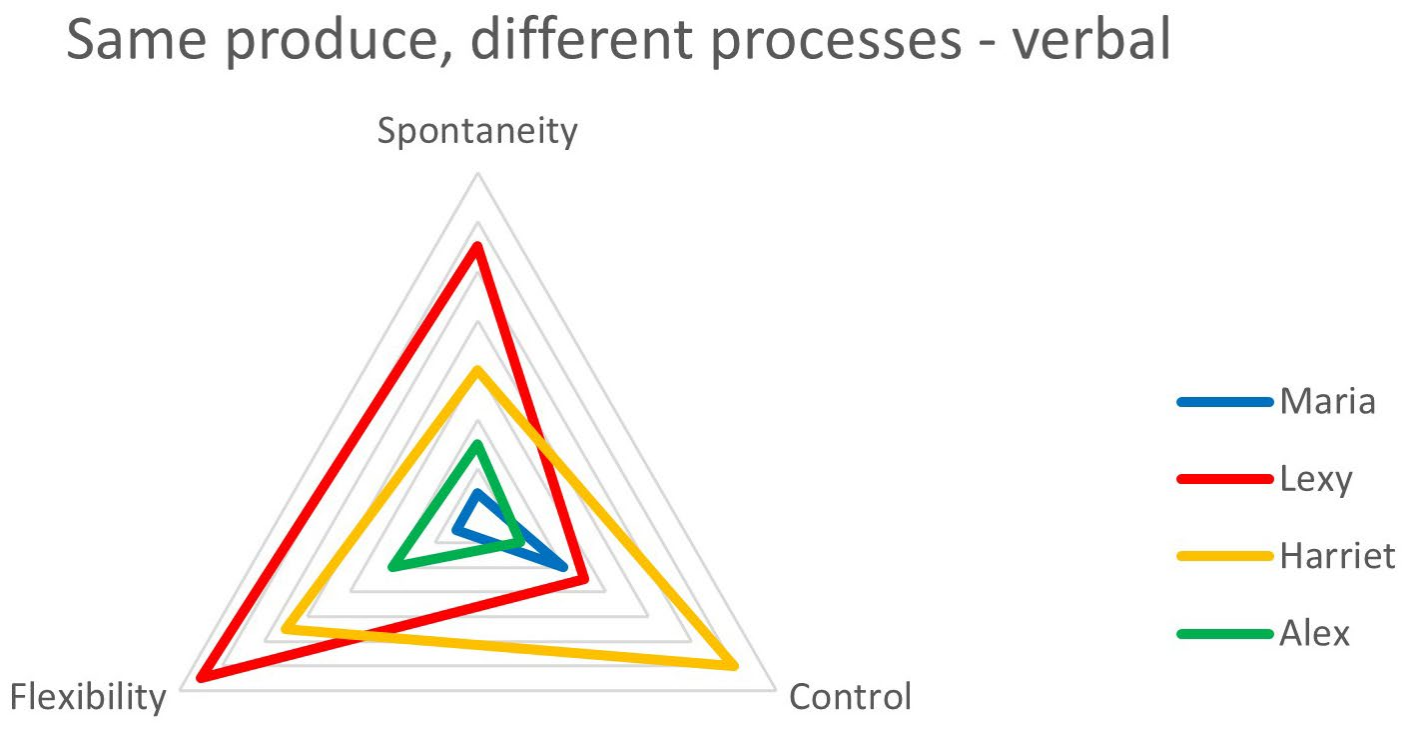
Four children who all scored 3 for originality in the AUT took different approaches to their creativity.

**Figure 6:**
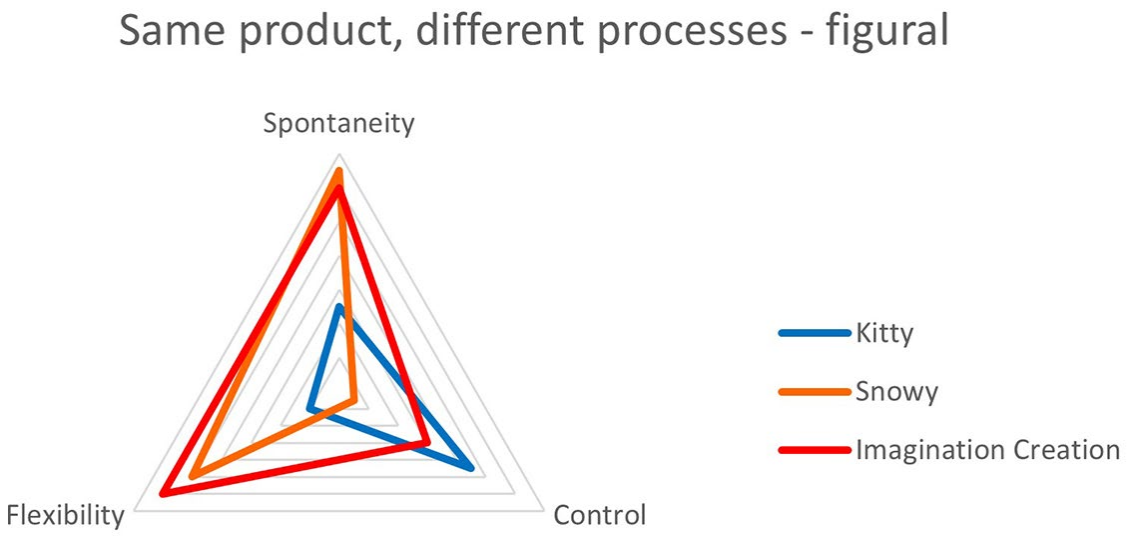
Three children who all scored 4 for originality in the figural tests took different approaches to their creativity.

An alternative way of looking at these data was to start with the behavioral outcome (the lab test product) and compare qualitative strategy (the reported creative process), i.e., begin with the quantitative and then look at the qualitative findings: was there evidence that children who achieved the same test scores did so by similar means? The illustrations below are for originality in the AUT (Figure 5) and originality in figural tests (Figure 6), two measures which showed no significant overall correlation with qualitative rank scores. These analyses rely on the chance outcome that four children all scored the same for AUT originality (a score of 3, just above the mean of 2.93). In the figures below, each colored line represents a child and shows their relative positions on axes of spontaneity, control and flexibility (highest scores on the outside). It suggests that, although their output was equivalent, the process by which it was produced was different for each of them. It also demonstrates how considering only products presents a partial picture.

A similar finding is shown in Figure 6, this time for originality in figural tests. Again, some children achieved identical scores for this measure: three scored 4, just below the mean of 4.15. Once again, their approaches appeared distinct.

#### 4.2.2. Summary

Triangulation findings showed no correlation between measures derived from real world creativity and quantitative lab-based creativity tests. Of the many possible reasons we have suggested, we think the most likely explanation is that the respective tests are measuring different processes. Correlating qualitative creativity measures with lab-based creativity tests showed, in line with predictions, highly significant correlations between spontaneity and AUT fluency and flexibility and consistently negative relationships between control and all quantitative creativity sub measures. The idea of control having negative effects on creativity was explored further by comparing performance of the most spontaneous with the most controlled children. The results showed that for fluency and flexibility control did indeed seem to impede creativity. The final analyses add depth to the qualitative findings that individual children deploy EC differently in their creativity. Looking at qualitative findings in isolation, we might conclude that EC differences would result in very different creative outcomes. Here however, we show that big differences in EC deployment can result in *the same* creative outcome.

## 5. Discussion

### 5.1. What did the individual studies show?

This paper has described three sets of findings relevant to the relationship between EC and creativity. The first were from quantitative lab studies of both constructs. Here, we found generally non-significant correlations. The lack of consistent relationships, positive or negative, between inhibitory control and creativity measures makes it difficult to help resolve the conflicting evidence base (Edl et al., 2014; Mayseless et al., 2015; Radel et al., 2015). In the few cases where there were significant relationships (as between verbal WM and AUT originality), these were only seen in one of the two verbal divergent thinking tests. It is possible that the creativity tests were not picking up a single construct. As mentioned, to reliably detect a correlation of 0.3 (at 80% power and a probability of 0.05) the study would have required a sample of 84, so being underpowered is a possible explanation.

The second set of findings came from a qualitative study of children’s creativity in a real-world setting which used stimulated recall to prompt children’s retrieval of the thought processes involved in their creative work. The analysis found that children’s descriptions fell into three broad themes: spontaneous processes (ideas arising unbidden, unprompted associations), control processes (planning, evaluating, strategic approaches) and processes which described the balance between these two extremes. These processes can be characterized as those that occurred outside of executive control, those that were tied to executive control and those that determined the extent of executive involvement.

These findings represented progress in answering one of our main research questions, i.e., to what extent are there individual differences in the role that EC plays in children’s creativity? The answer based on this evidence seems to be ‘a great deal’. The analysis demonstrated wide individual variation both in the extent to which children naturally tended to a more or less EC-led approach in their creativity (i.e., a trait difference in spontaneity vs. control) and in their ability to modulate the extent of that deployment (a trait difference in flexibility). In addition, there was much variation in the deployment of EC in creativity depending on context, task demands and domain (i.e., state differences). The differential involvement of EC in the creative process points to the insufficiency of quantitative tests alone for an education relevant skill like creativity.

### 5.2. What did triangulation achieve?

The third set of findings came from bringing together both data sets. Findings here showed there was no correlation between the primary qualitative dimensions and EC test performance. We think the most likely explanation is that ‘control’ as presented qualitatively describes a much broader set of activities than the more granular and specific processes represented by EC measures in lab tests (Astle and Scerif, 2009). This explanation comes with the caveat that triangulation involved (as intrinsic to the approach) small participant numbers. Triangulation also sought evidence of the relative effectiveness of different creative approaches (i.e., the extent to which creativity is control-led, spontaneity-led or flexible). To test the theory that too much control can be detrimental to divergent thinking (Radel et al., 2015), we looked at whether qualitatively derived control processes were negatively associated with quantitative creativity measures. While most of the correlations were indeed negative, they were not consistently statistically significant – for fluency and originality in the Just Suppose test, they reached significance, while in the AUT (fluency and flexibility) they did not. Associations with figural sub-scores were weak and not significant. The general notion that the influence of control on creativity can be a negative one aligns with evidence from other sources (Limb and Braun, 2008; Carson, 2011; Chrysikou et al., 2014; Radel et al., 2015; Beaty et al., 2016) though the dissimilarity of findings for different creativity tests still needs explaining. Just Suppose is a task based in an imaginary situation (‘Imagine that clouds had strings attached to them …’) whereas the AUT is based on an everyday, familiar object (a pencil, a plastic bottle) which perhaps makes it easier to solve through a controlled, strategic approach.

By contrast, spontaneity and flexibility were both highly positively correlated with fluency and flexibility in the AUT but showed no significant relationship with any sub-scores in Just Suppose or the figural tests. Again, the general notion that flexibility in particular is positively associated with creativity is supported by previous research (Nijstad et al., 2010; Zabelina and Robinson, 2010b; Vartanian et al., 2020) though the reasons for the inconsistencies in different tests are not immediately obvious. Perhaps the different starting points of divergent thinking could also have been relevant here; an ease with tapping into memory and sensory processes might have been helpful in the concrete world of the AUT but could have foundered when confronted with an imaginary scenario. The account of the role of EC in creativity might be one that crucially depends on the details and specific requirements of each task; this puts the onus on researchers to specify and characterize creativity task types and indicate the relevance of EC to each.

### 5.3. Implications for creativity

Deciphering and measuring the differences between lab creativity and real-world creativity presents a pressing and difficult problem for psychology and neuroscience, one compounded by the many specific difficulties of studying creativity, such as time, repeatability, spontaneity, space, and movement (Abraham, 2018). For example, while many argue that creativity can happen in ways that are either deliberate or spontaneous (Dietrich, 2004) “it is clear that when we assess creativity under lab conditions, we are mainly assessing deliberate forms of creativity” (Abraham, 2018, p. 48).

The triangulation results provide evidence that too much control can have negative consequences for creativity, specifically for fluency and flexibility. This is important in the context of the current enthusiasm for training EC. Given the accepted wisdom that EC improvements are unassailably a good thing (Huizinga et al., 2006; Diamond and Lee, 2011; Diamond, 2012, 2013; Diamond and Ling, 2016; Peters, 2020), it is especially important to question this assumption and present evidence of previously unconsidered negative side effects.

Whilst there is some evidence that particular approaches to creativity might generally be successful, it is important to remember that there is a great deal of individual variation, as evidenced by very diverse approaches which can result in identical scores. In fact, there is a danger that looking at overall results can obfuscate individual-level information. This can be a problem with certain types of mixed method triangulation (Bryman, 2007; Hesse-Biber, 2010). Using group-level results such as averages can mean that results, whilst true for the group, are not true for any individual within it. Attention should be paid to individual strategies and approaches since there seem to be many means by which creative goals can be achieved – *in extremis*, every individual might have their own unique approach. This has implications too for training creativity. In the considerable body of evidence which supports the idea that creativity can be trained (Scott et al., 2004; Zabelina and Robinson, 2010a; Gregory et al., 2013; Runco, 2014) are approaches which emphasise both EC-heavy approaches [e.g., using SCAMPER (Michalko, 1991): Substitute. Combine. Adapt. Magnify. (re)Purpose. Eliminate. Reverse] and those which emphasize the spontaneous nature of creativity, by creating environments which ‘let it happen’ (Runco, 2014).

### 5.4. Methodological implications for educational neuroscience

Determining the brain basis of any complex cognitive phenomenon hinges on understanding the components which underpin it. Most subjects of interest to educational neuroscience – maths ability, learning to read, understanding scientific concepts, creativity – fall into the complex cognition category. Added to this are the complex environment of the classroom and the complexity of potentially high levels of individual variability. A process-focused quantitative approach will only take us so far. Qualitative research, with its focus on process rather than product, has potential value in informing cognitive theories and mixed methods are increasingly seen, alongside quantitative and qualitative approaches, as ‘a third major research paradigm’ (Johnson et al., 2007, p. 112; Morgan, 2007; Tashakorri and Teddlie, 2007; Tashakkori and Teddlie, 2009, 2010; Creswell and Clark, 2011; Hesse-Biber and Johnson, 2015; Johnson et al., 2017). The adoption of mixed methods has seen significant growth in the last 20 years (Schwandt and Lichty, 2015), its chief motivation being a recognition that all methods have limitations and weaknesses and that combining different approaches “increases the likelihood that the sum of the data collected will be richer, more meaningful, and ultimately more useful in answering the research questions” (Preskill, quoted in Johnson et al., 2007, p. 121). The approach, for some, represents an enthusiasm and belief that science needs to progress beyond the limitations of single method approaches.

The methodological approach to any research depends on the questions being asked (Tashakkori and Creswell, 2007). At one extreme are questions which can only be answered qualitatively (‘How did your first day at secondary school feel?’); at the other, are those demanding a quantitative approach (‘How quickly can children react to a new visual stimulus?’). Most research questions of interest to educational neuroscience relate to neither extreme but to multifaceted constructs in unique individuals in complex environments, with performance depending on a range of situational, motivational, and emotional factors as well as cognitive ones. To gain traction on these complex questions, we think that educational neuroscience might benefit from an open-minded and experimental approach to its methodological choices.

## Data availability statement

The datasets presented in this article are not readily available because Consent was based on use in PhD dissertation and related publications. Requests to access the datasets should be directed to croger05@mail.bbk.ac.uk.

## Ethics statement

The studies involving human participants were reviewed and approved by Departmental Ethics Committee of Birkbeck’s Department of Psychological Sciences. Written informed consent to participate in this study was provided by the participants’ legal guardian/next of kin.

## Author contributions

MT and AT contributed to PhD supervision. CR conceived and designed all studies, with input from MT and AT. JM contributed to the design of Study 1, programmed the Flanker task, collected the executive functions data and pre-processed the Stroop and Flanker tasks. CR performed all the analyses and wrote the manuscript. MT contributed to manuscript revision. All authors contributed to the article and approved the submitted version.

## Funding

This research was funded by the Economic and Social Research Council, United Kingdom (Grant Numbers: 1649266 and 1788414).

## Conflict of interest

The authors declare that the research was conducted in the absence of any commercial or financial relationships that could be construed as a potential conflict of interest.

## Publisher’s note

All claims expressed in this article are solely those of the authors and do not necessarily represent those of their affiliated organizations, or those of the publisher, the editors and the reviewers. Any product that may be evaluated in this article, or claim that may be made by its manufacturer, is not guaranteed or endorsed by the publisher.

## Footnotes

^1^https://gorilla.sc/
